# Unlocking the potential of essential oils in aromatic plants: a guide to recovery, modern innovations, regulation and AI integration

**DOI:** 10.1007/s00425-025-04724-y

**Published:** 2025-05-25

**Authors:** Sneha Nayak, Roopa B. Hegde, Abhishek S. Rao, Ramaprasad Poojary

**Affiliations:** 1 Department of Biotechnology, NMAM Institute of Technology, Nitte (Deemed to be University), Nitte, Karnataka 574110 India; 2 Department of Electronics and Communication Engineering, NMAM Institute of Technology, Nitte (Deemed to be University), Nitte, Karnataka 574110 India; 3 Department of Information Science and Engineering, NMAM Institute of Technology, Nitte (Deemed to be University), Nitte, Karnataka 574110 India; 4 School of Engineering and IT, Manipal Academy of Higher Education, Dubai Campus, P O Box no, 345050, Dubai, United Arab Emirates

**Keywords:** Essential oils, Distillation, Tissue culture, Business innovations, Aromatherapy, Artificial intelligence, Machine learning

## Abstract

**Main conclusion:**

Essential oils recovered from aromatic plants hold tremendous potential across diverse fields, which include therapeutic, industrial, and technological domains. Integrating advanced recovery techniques, regulatory frameworks, and AI-driven innovations can significantly elevate their sustainable production and application.

**Abstract:**

There is a growing need for essential oils (EOs) due to their therapeutic and aromatic benefits, they have been explored across the globe. This comprehensive guide tries to give a glimpse of the skyrocketing potential of EOs by highlighting the advancements in recovery techniques, modern innovations, regulatory frameworks, and its integration with modern technologies like artificial intelligence (AI). The review highlights the importance of quality control in ensuring purity of EOs, discusses various extraction methods, and explores emerging technologies like supercritical fluid extraction and microwave-assisted extraction for successful and quick recovery of EOs with minimal energy requirements. The guide also delves into modern innovations such as encapsulation techniques and nanoemulsion formulations, expanding the scope of essential oils in industries like cosmetics, personal care, and healthcare sector. Addressing the regulatory aspect, the review emphasizes on the standardized quality testing, labeling, and safety considerations necessary for EOs to reach the market. Further, the paper explores the applications of EOs in business innovations such as preservatives to extend shelf life and aromatherapy for pain reduction. Overall, this guide provides valuable insights into the future of EOs, showcasing the possibilities enabled by advancements in techniques, innovations, regulations, and AI integration, benefiting researchers, industry professionals and public.

## Introduction

Essential oils (EOs) are complex concentrated mixture of volatile substances synthesized in aromatic plants by secondary metabolism under the influence of biotic or abiotic stress conditions (Asadollahei et al. 2023). EOs is widely explored in pharmaceutical industries for its benefits due to the presence of numerous biologic active agents (Dhama et al. 2021). Over the past decade more than 3000 EOs have been potentially explored and characterized both physically and chemically and around 150 are produced at industrial scale (Zhang and Piao 2023). Various parts of the plants like leaves (Aatiaoui et al. 2022), petals (Fereidani and Uctug 2023), seeds (Aly et al. 2022), stem (ElAchaouia et al. 2023), rhizome (Cvetkovic et al. 2022), and roots (Kılıç et al. 2023) have been exploited for oil production through processes like hydrodistillation (HD) (Shaw et al. 2023), microwave assisted hydrodistillation (Mohammadhosseini et al. 2023), ultrasound assisted hydrodistillation (UA-HD) (Jadhav et al. 2023), cold pressing (Prestes et al. 2023) and solvent extraction (Zanotti et al. 2023). For instance, Rhizome of ginger (Munda et al. 2018) and petals of rose (Katekar et al. 2022) has been explored for aromatic oil recovery. The main reason for exploring plants for these precious volatile compounds (terpenes, esters, phenols, alcohols and ketones) is due to the fact that these molecules are present in extremely small quantities as multiple metabolites and its challenging to replicate synthetically due to complexity and diversity (Das et al. 2023).

The biologic role of EOs production in aromatic plants is either to protect from pests/to attract pollinators (Feng, et al. 2022). Over the years, apart from use of EOs as herbal medicines they have been explored in other industries like food (Tsitlakidou et al. 2023), perfumery (Antoniotti et al. 2022), and cosmetics (Rasheed et al. 2021). Roots of *Carlina acaulis* have been explored for insecticidal EOs production (Spinozzi et al. 2023) and leaves of *Calamintha* as anticorrosive agents (Aatiaoui et al. 2022). Thymol, an active ingredient in *Thymus vulgaries* is used for treatment of menstrual cramps (Santana de Oliveira et al. 2023) and EOs from *Deverra spp.* has wound healing properties (Santana de Oliveira et al. 2023). Limonene monoterpenes a key constituent of citrus EOs is extensively explored for flavor and aroma business (Soni 2025). Similarly, eugenol from clove finds applications in dental pain relief (Kim et al. 2023), menthol from peppermint find application in relieving headaches (Abo-Elghiet et al. 2025) and geranial from lemongrass finds application in antiaging skincare products (Kusuma et al. 2024).

The recovery of EOs from plant parts is a crucial step in the production of EOs. Conventional methods, such as steam distillation (Vargas et al. 2022) and its variants (microwave assisted and ultrasound assisted systems) and cold pressing (Bento et al. 2020), are widely utilized and have stood the test of time. Steam distillation involves passing of hot steam through plant parts, followed by extraction, and condensation into a liquid form. Cold pressing, on the other hand, was primarily used for obtaining precious oils from citrus peels. In the recent years, advancements in technologies, including solvent extraction (Cvetkovic et al. 2022), supercritical CO_2_ fractionation (Zanotti et al. 2023), and molecular distillation (MD) (Idárraga-Vélez et al. 2023), have proven more efficient and selective methods for recovery of EOs or EO extracts. Due to the numerous therapeutic benefits of EOs, including sedatives (Yigit and Kocaayan 2023), antiplasmodic (Marghich et al. 2023), hypnotic (Tang et al. 2023), analgesic (Yigit and Kocaayan 2023), neuroprotective (Soliman et al. 2023), antimicrobial (Kaboudi et al. 2023), antioxidant and cytotoxic (Al-Zereini et al. 2023), anti-inflammatory (Righi et al. 2023), antiallergic (An et al. 2023), antitumour (An et al.^2023^) and immunoregulatory properties (Tang et al. 2022) for promoting overall well-being exploration of EOs have increased over the years.

Recent reviews have explored the use of EOs as nutraceuticals (Jan et al. 2024), nutritional agents (Pezantes-Orellana et al. 2024), healthcare and pharmacological applications, such as stress reduction (Gusmão 2024), arthritis treatment (Mahajan et al. 2023), and COVID-19 management (Mahajan et al. 2023). However, there is no much information on crucial topics like EO characterization, regulatory requirements for EO sales, and the use of artificial intelligence (AI) to identifying potential plant sources, developing antimicrobials, and improving EO quality. Therefore, this review aims to provide a comprehensive overview by highlighting recent business innovations in food and healthcare and also discusses important aspects of EO recovery, characterization, and AI integration, offering a valuable point resource for those interested in EO research.

Finally the production and sale of EOs are subject to various regulatory requirements worldwide to ensure consumer safety and high product quality (Wei et al. 2023). These regulations include aspects such as labeling (Sattariazar et al. 2023), purity standards to be maintained, and good manufacturing practices. Adherence to these guidelines is crucial to maintaining the integrity of EOs and preventing adulteration. In addition, quality control measures with robust techniques including gas chromatography-mass spectrometry (GC–MS) (Joshi et al. 2022), FTIR (Kaboudi et al. 2023), GC-EAG (Yigit and Kocaayan Jan. 2023) and organoleptic evaluation (Zhang et al. 2023), are employed to verify the chemical composition and authenticity of EOs.

The field of EOs is continually evolving, driven by on-going research and tremendous technological advancements. Modern business innovation strategies have expanded the applications of EOs beyond traditional practices (Yan et al. 2019). For instance, EOs are now being incorporated into personal care products (Gad et al. 2023), food and beverage formulations (Hadian et al. 2023) and even household cleaners (Kumar et al. 2023). Furthermore, research efforts have focused on exploring the antimicrobial potential of EOs, for use as natural preservatives (Islam et al. 2023), and checking their efficacy in addressing various health conditions.

As the demand for EOs continues to rise, sustainable practices in cultivation, analysis of plant growth, harvesting, selection of processing techniques and shelf life analysis of EOs become paramount (Valle-García et al. 2023). Some of these aspects could be addressed by inception of AI and machine learning (ML) (Yap et al. 2021). Recently, smartphone-based handheld Raman spectrometer and ML tactics have been explored for EO quality evaluation and Deep learning (DL) model for better classification and bioactivity prediction in EO producing plants from Egypt (Lebanov and Paull 2021). Therefore, amalgamation of recent technological advancements could help in preservation of biodiversity, responsible sourcing tactics, and fair trade practices to ensure the long-term availability of plant materials for EOs production. Furthermore, efforts to educate consumers about sustainable consumption of EOs and the importance of supporting ethical and environmentally friendly practices are necessary for its successful use.

This review paper provides an in-depth examination of EO production strategies in plants, highlighting the most recent advancements in the field. Emphasizing the importance of oil characterization to meet international quality standards, and its exploration in innovative applications is emphasized. Furthermore, the potential of AI and ML models to predict biologic active ingredients is explained. Moreover, insights into plant age for optimizing EOs yield which could be beneficial to the farmers to gain better profits is stressed upon. The review paper thus offers a concise overview of the roadmap for EO production, advancements in production strategies, and the integration of AI and ML for boosting farmers profits.

**Fig. 1:**
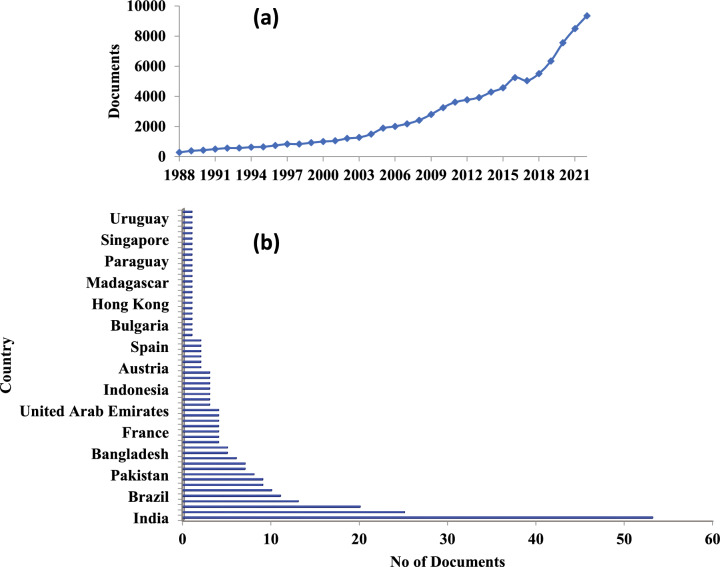
Papers published on “Essential oils” between 1988 and 2022 (**a**); and on “Essential oils as therapeutics papers country wise” between 2002 and 2022 (**b**), as per recent search in Scopus Database (accessed on 27/06/2023)

**Fig. 2:**
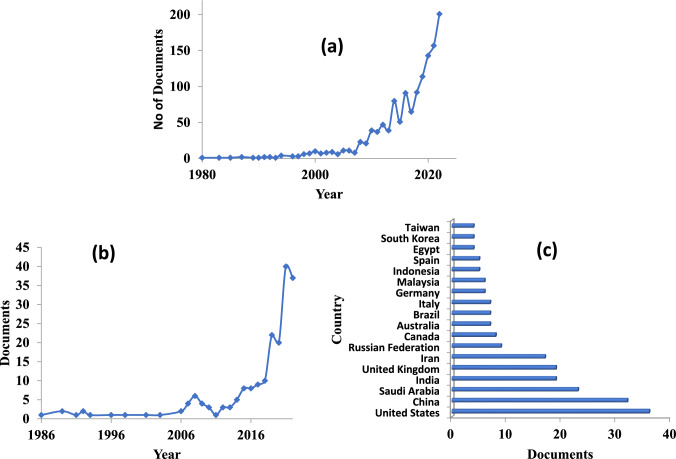
Papers published on “Essential oil production strategies” between 1980 and 2022 (**a**); and on “Artificial Intelligence and essential oil” between 1986 and 2022 (**b**), and country wise documents on “Artificial Intelligence and essential oil” details as per recent search in Scopus Database (accessed on 27/06/2023)

A desk review of the Scopus database carried out with the keyword “Essential oils” between 1988 and 2022, shows, rapid increase in the papers published over the years with 95,641 documents (Fig. 1A), while research on “search for “Essential oils as therapeutics” study from 2002 to 2022 showed only 181 documents showing the untapped potential of research avenues in this area (Fig. 1B). India topped the list with 53 documents and USA stood 2nd with 23 documents, understanding the need for further research in this area. However, search results on “Essential oil production strategies” between 1980 and 2022, shows 1305 documents where USA tops the list with 239 documents followed by China with 155 and India with 98 documents (Fig. 2A) and “Artificial intelligence and essential oil” between 1986 and 2022, shows 196 documents (Fig. 2B). This proves that more research in the role of AI and ML approaches in predicting the type, age and processing techniques required for aromatic plants. This could benefit the farmers for getting better yield of EOs and maximizing profits. Therefore, business innovations and producing superior quality EOs would help in exports of EOs internationally.

## Essential oil and their production strategies in plants

**Fig. 3:**
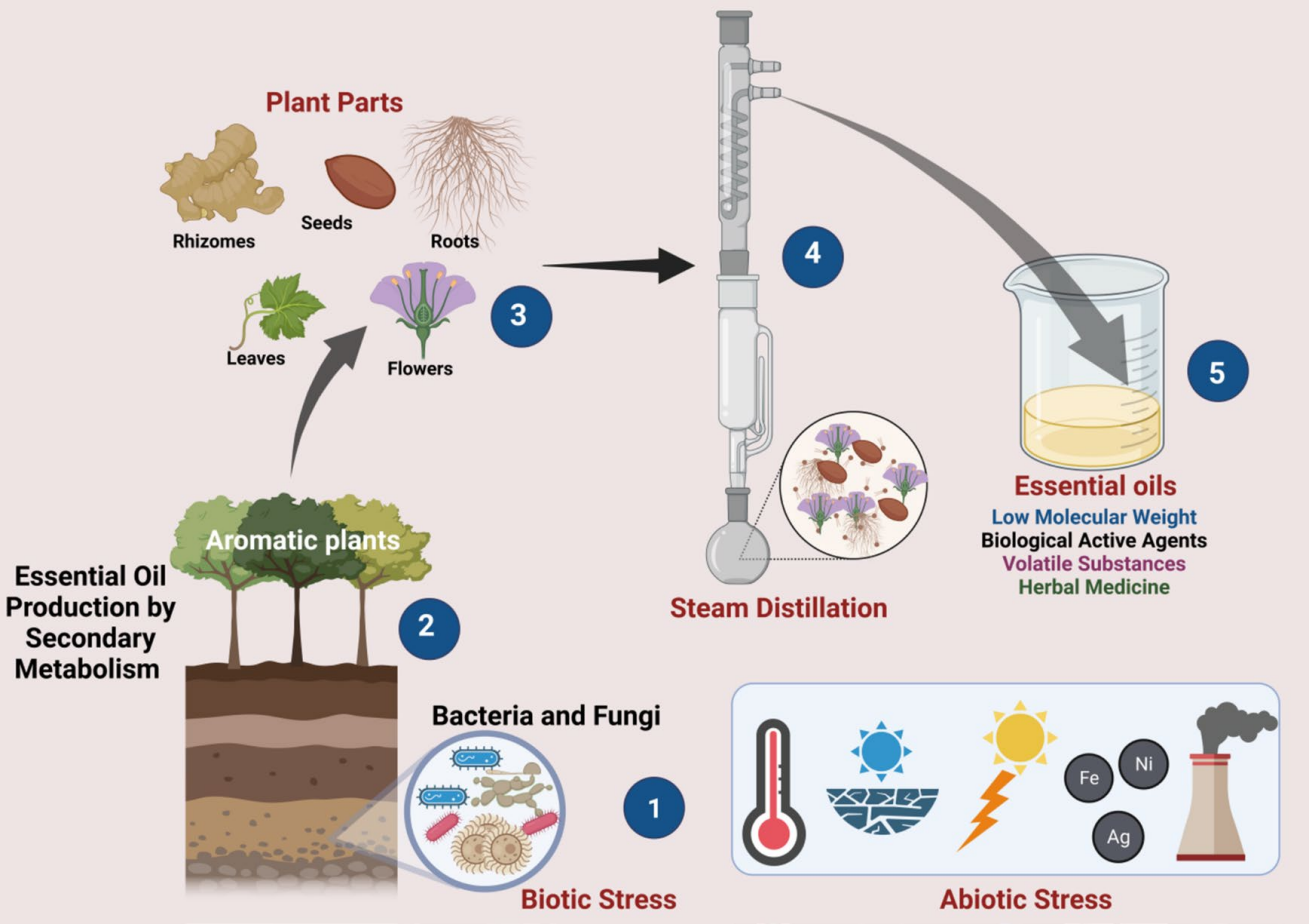
Overview of secondary metabolism in aromatic plants under various biotic and abiotic stress conditions

Since plants do not have the capability to escape from its predators unlike animals, plants have evolved several mechanisms to defend against potential threats (Zhang 2023). One such mechanism is production of odorous secondary metabolites either to attract pollinating insects or as defensive mechanisms against stress conditions (Wink 2018). These odorous secondary metabolites indirectly help in increasing the ecological interactions with pollinators. In contrary, primary metabolism in plants is necessary for the growth and development of a plant and does not include secondary metabolite synthesis (Tariq et al. 2023). The quantity of quality of secondary metabolites production in plants is ascribed not only to the genetic make of the plant but also to the growth stage of the plant as well as environmental conditions (Verma and Shukla^2015^). For instance, there are quite a few evidentiary literature reports which indicates temperature, drought conditions, sunlight, heavy metals as considerable abiotic stress conditions for the production of EOs (Fig. 3). Shift from primary to secondary metabolism in plants as a crucial adaptive mechanism is coordinated by regulated processes like signal transduction, gene regulation, metabolic reprogramming and enzymatic control which is followed by secondary metabolite production and its value addition. Signal transduction involves the activation of intracellular pathways by biotic and abiotic stress leading to ROS, calcium ion influx and phytohormonal changes in plants. Furthermore, stress signals are known to upregulate the genes responsible for EO production (Olalere et al. 2024). For example, jasmonic acid is a widely explored stress signal for terpenoid biosynthesis (Khan et al. 2024). As metabolic reprogramming strategy in plants divert the primary metabolism related pathways (glycolysis and krebs) to secondary metabolism pathways thus producing precursors for EO synthesis thus helping in upregulation of terpenoid pathways (Samsami and Maali-Amiri 2024). Similarly, cytosolic mevalonate pathways produce sesquiterpenes and plastidic methylerythritol phosphate pathway produce monoterpenes like limonene (Singh et al. 2024). In addition, production of phenolic compounds is enhanced by shikimic acid pathway (Li et al. 2024). Finally, enzymes like terpene synthases catalyses the formation of mono and diterpenes and phenylalanine ammonia lyase regulate phenylpropanoid biosynthesis and these EOs are further accumulated in specialized structures like glandular trichomes, secretory cavities and resin ducts for defensive action (Manfron et al. 2021).

In *Ocimum tenuiflorum* plant cold stress (15 °C) and drought stress (26° ± 2 °C @ RH of 65 ± 5%) for 5 days stimulated the production of EOs (eugenol, methyl eugenol, and β-caryophyllene) (Nguyen et al. 2022). The biosynthesis of secondary metabolites could eventually be affected by the growth of medicinal plants in areas with high levels of heavy metal pollution, leading to observable changes in the amount and quality of EOs along with added benefit of remediation of polluted sites (Asgari Lajayer et al. 2017). Literature reports on Genetic studies have demonstrated that heavy metal exposure can enhance the expression of genes involved in EO synthesis, offering the dual benefit of phytoremediation and increased EO production (Hubai and Kováts 2024).

**Fig. 4:**
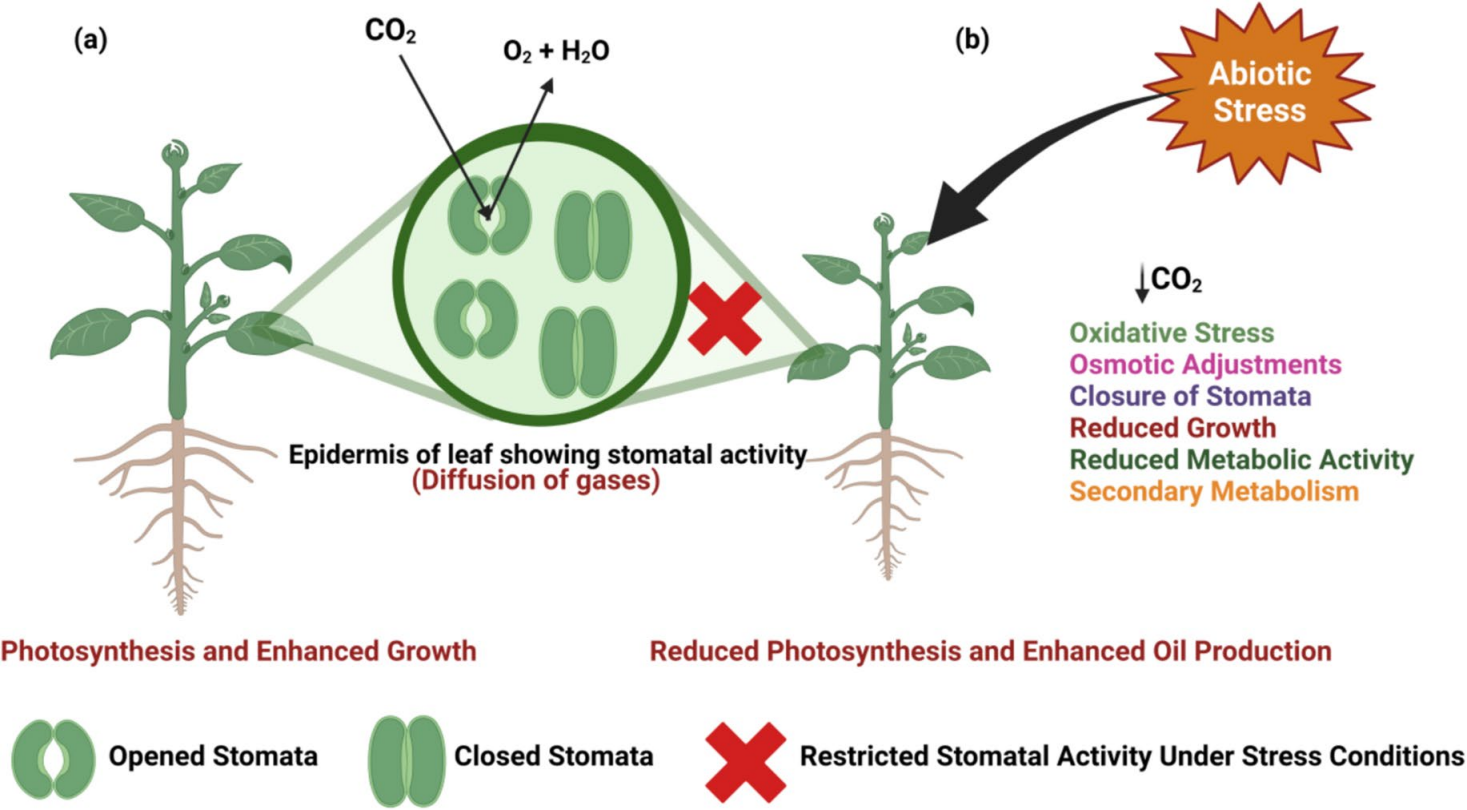
Overview of **a** photosynthesis and enhanced growth and **b** negative impact of abiotic stress on photosynthetic activity of plants due to restricted stomatal activity

Abiotic stress conditions also create an imbalance between reactive oxygen species (ROS) synthesis and antioxidant mechanism in plants thus resulting in increased oxidative stress conditions (Fig. 3). Further, negative impact on growth and reduced photosynthetic activity due to degradation in photosynthetic machinery reduces the primary growth in plant by restricting the stomatal activity (Fig. 4). This approach helps the plants to shift to secondary metabolism related pathways which helps in enhancing the yield of EO production (Kumar et al. 2022).

Biotic stress on the other hand involves the symbiotic relationship between microbiota (Bacteria and fungi) and the aromatic plants (Fig. 4). *Bacillus subtilis* causes systemic resistance in plants directly through the production of secondary metabolites, hormones, and antioxidants as well as through supporting the plant’s natural defenses against possible infections. However, indirect mechanism could be through solubilisation of soil pathogens, and production of siderophores which down regulates the growth of pathogens (Hashem et al. 2019). Therefore, both direct and indirect mechanisms would help in enhancing the yield of EO in aromatic plants. Mechanistic Insights and overview on influence of abiotic stress conditions on EO production is depicted in Table 1.

**Table 1:** Mechanistic Insights and overview on influence of abiotic stress conditions on essential oil production

Botanical name	Common name	Oil name	Stress condition	Mechanism	References
*Tagetes minuta L*	Marigold	Tagetes oil	Drought salinity and heavy metals	*Induces drought stress, which activates secondary metabolism in plants, leading to increased biosynthesis of essential oils as a defense response by ROS formation and growth deterioration *Associated pathways: mevalonic and shikimic acid *Decrease in photosynthetic activity	Kumar et al. (2022)
*Satureja hortensis L*	Savory	Savory essential oil	Water deficit and bio stimulants like amino acids	*Reduction of relative water content * Antioxidarive and defence enzymes involved: Superoxide dismutase (SOD), Catalase (CAT), and Peroxidases (PODs) *Reduction in photosynthesis rate and dry matter production	Rezaei-Chiyaneh et al. (2023)
*Chamomilla recutita L*	German chamomile	Blue oil	Acute drought conditions	*Impaired capacity to water uptake *Promotes the closure of stomata to ensure water efficiency *Activation of antioxidative defense system	Punetha et al. (2023)
*Artemisia dracunculus L*	Tarragon	Tarragon essential oil	75% of light intensity and moderate salinity (60 mM)	*Closure of stomata and inhibition of cell growth *Ion cytotoxicity and slowing down of metabolic process	Mohammadi et al. (2023)
*Lippia alba*	Verbenaceae	Lippia alba essential oil	Salinity and *mycorrhizal* association *(Fuscutata heterogama* and* Claroideoglomus etunicatum*)	*Defence mechanism against salinity *Carbohydrates reallocated to shikimic acid pathway	Neto et al. (2023)
*Mentha Piperita*	Peppermint	Peppermint oil	Plant hormone: brassinosteroids (1 mM) Nacl: 60 mM	*Enhanced detoxification of reactive oxygen species by plant hormone alleviated NaCl-induced oxidative stress *Modulated antioxidant enzyme activity, including superoxide dismutase (SOD) and catalase (CAT), thereby enhancing reactive oxygen species (ROS) detoxification	Fathi et al. (2023)
*Stevia rebaudiana Bertoni*	Stevia	Stevia oil	*Drought stress conditions *Affirmative role: Melatonin (100 µM) and TiO_2_ NPs (10 mg/L)	*Stomata regulation *Osmotic adjustments	Sheikhalipour et al. (2022)

## Innovative techniques in recovery of essential oil—green methods

Over the years a lot of innovative techniques have been tried and tested for recovery of EOs. Among the methods, distillation is the widely and most frequently accepted physical technique used for the recovery of EOs. Further, advancements in distillation types, extraction options have also been tried for maximizing the EO yields.

### Distillation and its types

Prior to distillation, plant parts rich in oil are typically dried and appropriately crushed to break open the oil sacs and expose the overall surface area for effective oil release. Below are the quick understandings on various distillation methods that are frequently used for the recovery of EOs.

#### Hydrodistillation

Hydrodistillation (HD) is also known as direct heating distillation involves the use of prepared plant materials directly with water in a boiler and application of heat for oil recovery. Although the majority of the constituents of EOs typically have boiling points between 150 and 300 °C, they can be evaporated using steam or boiling water at 100 °C because the combined vapor pressure of the two immiscible liquids will be equal to atmospheric pressure at a temperature lower than the EO’s vaporization temperature (Dalton’s law). Citrus peel dried using a tray dehydrator @45 °C, powdered and hydrodistilled achieved 9.98% of EO which were rich in sesquiterpenes, terpenoids and D-limonene (Shaw et al. 2023). The main disadvantage of this HD system is slow recovery of oil and requirement of close manual attention which is cumbersome.

#### Steam distillation

**Fig. 5:**
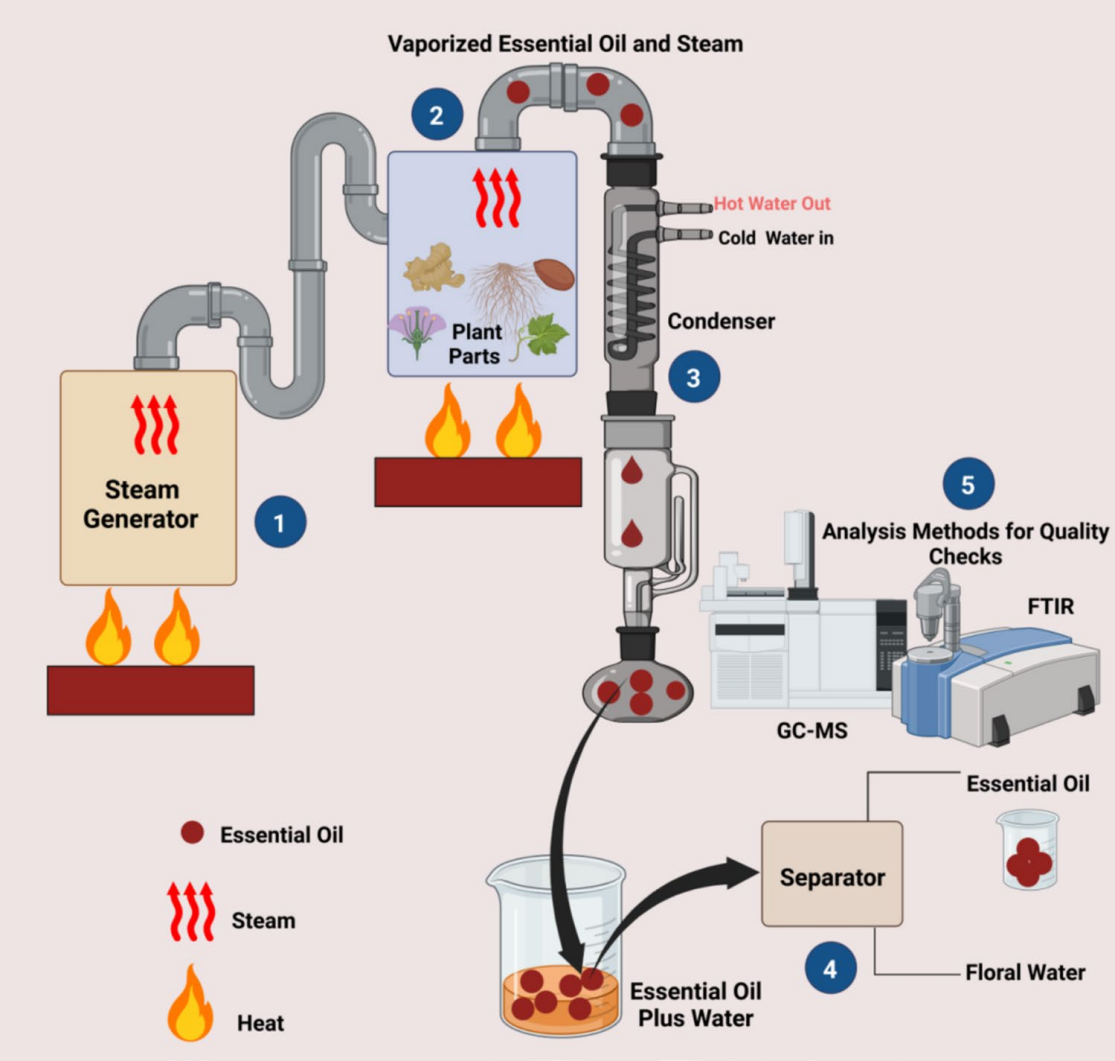
Roadmap of Essential oil recovery by steam distillation and need for analytical methods for oil quality checks

Steam distillation is a faster technology in comparison to HD. In this method a high-pressure steam is passed over prepared plant material which avoids the direct heating and minimizes thermal decomposition of EO components which have lower boiling points. Flowers of *Acacia mearnsii* were subjected to steam distillation at 2 bars, to recover 8-heptadecene as major component. Evaluation of EO was carried out by GC/FID, GC/MS, GC/O (Vargas et al. 2022). The roadmap of recovery of EO by steam distillation and need for analytical methods (like GC–MS/FTIR) for quality checks is depicted in Fig. 5.

#### Microwave assisted hydrodistillation

Microwave assisted hydrodistillation (MA-HD) has shorter extraction time, minimizes energy consumption by uniformly heating the sample It provides higher yields, improves the quality of oxygenated compounds, eco-friendly and is an economically viable option for EO recovery. MA-HD has better extraction potential in comparison with conventional hydrodistillation (slow process) as it directly heats the plant material thus leading to faster cell rupture and improved essential oil release. Optimization techniques like response surface methodology (RSM) have been explored by researchers for maximizing the yield of essential oil/improve the extraction efficiency. Optimization of parameters like microwave power utilized, liquid to material ratio used and extraction time set would be critical in improving the extraction efficiency. For instance, dried powder of *Rumex crispus* leaves showed maximum yield of oil (4.67 ± 0.02%) @534.89 W power, 23.48 min HD time and 4.5 ml/g liquid to material ratio. The extraction by MA-HD showed higher oxygenated compounds (α-santol and β-santol) in GC–MS analysis (Beyecha Hundie et al. 2023).

#### Ultrasound assisted hydrodistillation (UA-HD)

UA-HD also known as sonohydrodistillation is an innovative technique which couples extraction with sonication. The mechanism of extraction is through the process of cavitation bubble formation and its explosion by sonic waves in the liquid medium. Time required for extraction is also minimal with enhanced oil yield making this process a green and energy efficient approach. Use of UA-HD for essential oil recovery from clove buds and optimization of process parameters by Box–Behnken design (BBD) yielded 15.23% w/w essential oil at optimum conditions (extraction time of 30–50 min, solid loading of 10–30 g/500 ml water, power of 300–500 W and pulse interval of 20:40–40:20) (Jadhav et al. 2023).

##### Molecular distillation (MD)

MD, also known as short path vacuum distillation/thin film distillation, is a popular method employed in the cannabis industry to extract heat-sensitive constituents. By evaporating the oil in the presence of a wiper system under vacuum, the boiling point of the mixture is further lowered, leading to the formation of a thin film system. This film formation effectively reduces thermal stress and minimizes the duration of the entire process, thereby enhancing the efficiency of distillation. Consequently, this method proves to be cost-effective, requiring less energy for operation. Use of RSM to optimize the distillation of cannabis oil by thin film distillation system was tried. For the predicted optimal conditions of feed flow rate of 41.6 mL/min and condensation temperature of 75 °C the recovery efficiency of tetrahydrocannabinol was 93.4% without compromising on the quality (Reddy Sagili et al. 2023).

### Supercritical CO_2_ extraction (SC-CO_2_ Ex)

SC-CO_2_ extraction (SC-CO_2_ Ex) modernizes the extraction process by harnessing the exceptional properties of CO_2_ as an extracting solvent. CO_2_ serves as the ideal extraction fluid due to its remarkable qualities, including non-toxicity, safety, abundant availability, and eco-friendliness (Zaman et al. 2020). Unlike other liquids, supercritical fluids, such as CO_2_, lack surface tension and possess low viscosities. These characteristics enable them to penetrate even the tiniest pores of plant material that were previously inaccessible to conventional solvents/extraction methods (Manjare and Dhingra 2019).

One of the key advantages of SC-CO_2_ Ex is the exceptional stability it imparts to the essential oil extracts without compromising their inherent stability. This is a significant benefit, ensuring the preservation of the oils quality over time. Furthermore, SC-CO_2_ Ex demonstrates a wide-ranging affinity, not only for volatile components but also for non-polar components present in plant materials (Pambou 2017). Consequently, it proves to be an excellent choice when extracting EOs with diverse properties, all within a single extraction process. An exploration of the influence of different temperatures (40–60 °C) and pressures (100–300 bar) on the extraction of terpenes and piperamides from *Piper nigrum* L. by SC-CO_2_ Ex revealed noteworthy findings. Terpenes could be efficiently recovered in shorter durations, requiring less time for extraction. On the other hand, the recovery of piperamides necessitated the maintenance of higher pressure conditions. Leaf essential oils rich in thymol with enhanced antimicrobial properties have been extracted using supercritical technology using mild temoeratures (40–50 °C) and high-pressure conditions (100–300 bars) (Silva et al. 2021). These insights underscore the adaptability and control offered by SC-CO_2_ Ex, allowing for tailored extraction processes depending on the desired components (Luca et al. 2023).

### Tissue culture techniques for essential oils

To address the challenges posed by uncertain raw materials supply for EOs production, it is crucial to integrate modern biotechnology tools with traditional farming practices. Unavoidable factors such as climate change, natural disasters, and plant diseases have made achieving expected yields a difficult task. In addition to these challenges, geographic limitations and the sluggish growth of plants have posed significant obstacles to scaling up EOs production to meet industrial-scale requirements. To overcome this problem, plant tissue culture presents a viable solution by employing innovative techniques to design an optimal medium for shoot and root propagation through micropropagation. By simulating controlled growth conditions, plant tissue culture can enhance EOs yield by mimicking ideal growth conditions. Biotechnological intervention like plant tissue culture have been successfully used to boost secondary metabolite production unaffected by geographic or climatic factors (Duta et al. 2023). A general overview of Plant tissue culture techniques used, conditions maintained for EO production is shown in Table 2.

**Table 2:** Plant tissue culture techniques for EO production

Source	Explant used	Conditions maintained	Major findings	References
*Plectranthus amboinicus*		**Shoot multiplication**: Semi-solid Murashige and Skoog (MS) medium supplemented with 0.4 mg/L 6-benzylaminopurine (BAP) **Optimal rooting**: MS medium with half strength **Acclamatization**: Sterile peat-moss moistened with MS medium in glasshouse for 2 months	Media supplemented with higher concentrations of BAP or used in combination with gibberellic acid (GA_3_) produced abnormal plantlets	Arumugam et al. (2020)
*Carum copticum* L	Seedlings	**Callus induction**: MS medium supplemented with 4-μM benzyl amino purine and 1 μM 2, 4-dichlorophenoxyacetic acid **Culturing**: MS medium containing 4-μM BAP, 1-μM 2, 4-D, and different concentrations of chitosan with 100-mM NaCl	*Improved the production of target secondary metabolites through abiotic elicitors supplemented to the medium *Chitosan (20 mg/L) found to be most effective on phenol content with increased production of thymol and p-cymene (an favorable excipient for the pharmaceutical industry)	Razavizadeh et al. (2020)
*Salvia sclarea*	Seeds	**Shoot multiplication**: MS medium supplemented with 1.5 μM benzyladenine (BA) and 0.15-μM IBA (30 days) **Optimal rooting**: MS medium with 5 μM IBA	*Essential oil was rich in secondary metabolites (linalyl acetate, trans-caryophyllene and its oxide, a-copaene and germacrene D) *Extraction yield of sclareol from the blossoms was 1.5–2 g/100 g of dry mass *Micropropagation protocol resulted in plant propagation in 330 days and field cultivation with the first harvest in 450 days	Grigoriadou et al. (2020)
*Mentha piperita L*	*Apical meristems and nodal segments*	MS medium with the addition of 0.5 mg/dm^3^ indolyl-3-butyric acid	*Production of desirable odor-active compounds (OACs) like menthofurolactone and cis-carvone oxide and improved share in the plant aroma profile *Volatile organic compounds (VOCs) obtained from plants of different origin by GC–MS showed no significant differences in their qualitative composition	Łyczko et al. (2020)
*Acorus calamus*	Rhizome bud	**Shooting multiplication:** Murashige and Skoog’s medium supplemented with a combination of BAP (3.0 mg/l) and IAA (1.0 mg/l) **Optimal rooting**: MS medium supplemented with auxins	*Maximum contents of total phenolics, flavonoids and higher antioxidant properties in tissue culture raised plants as compared to field grown ones *Methanolic and acetone extract of rhizomes of micropropagated plants showed slightly elevated antibacterial activity against five bacterial pathogens	Babar et al. (2020)

**Fig. 6:**
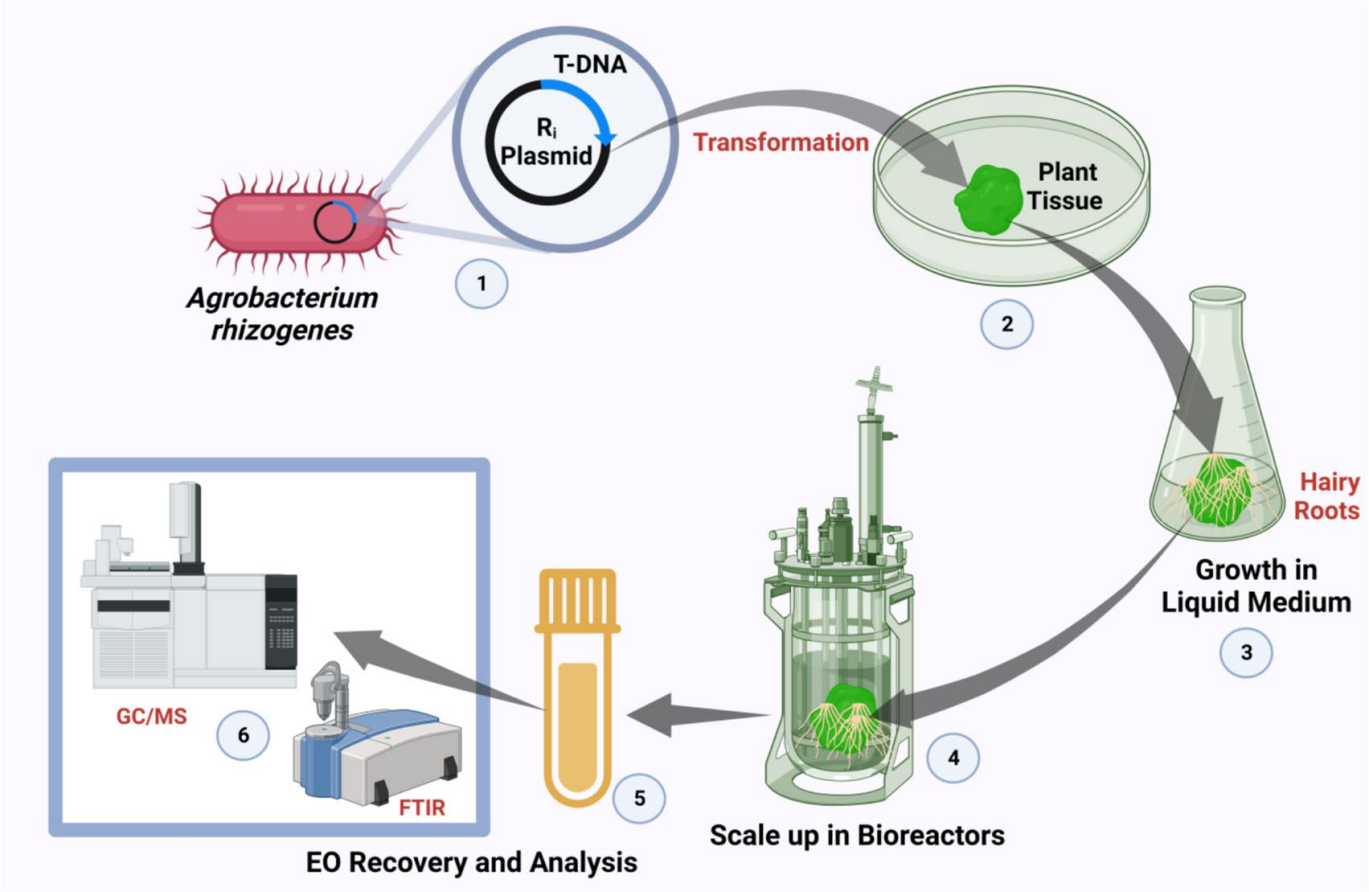
Overview of *Agrobacterium rhizogenes* mediated transformation of plant tissue for hairy root culture development and scale up in bioreactors for EO recovery

Implementing the hairy root culture technique, which involves infecting explants with specific strains of *Agrobacterium rhizogenes*, offers an additional strategy to boost EOs production and meet the demands of industrial-scale requirements. Hairy roots are genetically more stable and biologically dynamic making them highly dependable for generating active chemicals compared to traditional tissue culture methods. This culture system brings several advantages, including the requirement of minimal explant material and the ability to initiate metabolite production quickly. Moreover, hairy root cultures can be readily scaled up using bioreactors, enabling continuous and efficient EOs production (Chakravarty et al. 2021). Figure 6 illustrates the process of utilizing *Agrobacterium rhizogenes* mediated transformation to develop hairy root cultures in plant tissue, followed by their scaling up in bioreactors for EO recovery and quality assessments. The current progress in utilizing genetic engineering techniques in aromatic plants to enhance EO production has been limited so far. In-comparison with strong regulatory requirement and public scrutiny for GM food, non-food crops producing EOs while also regulated, may have more minor flexibility depending on their intended use therapeutic/industrial application. However, consumers demand for natural and eco-friendly products may pose a barrier to acceptance, thereby requiring clear labeling and transparent communication about genetic modifications in labels. However, there is on-going research worldwide focusing on this area. It has been acknowledged that genetic transformations play a crucial role in increasing EO yield and improving the quality of the desired product. Engineered plantlets have diverse applications, such as large-scale production of the desired product, enhanced resistance against natural stress conditions, improved disease resistance, and increased yield. Researchers have experimented with innovative plant breeding techniques, such as clustered regularly interspaced short palindromic repeats (CRISPR), to enhance oil crops for enhanced EO production (Li et al. 2024). However, the widespread acceptance of these technologies by the public remains a significant concern (Rahman et al. 2033). Important Genes like DXS (1-deoxy-D-xylulose-5-phosphate synthase), TPS (terpene synthase) and HMGR (3-hydroxy-3-methylglutaryl-CoA reductase) are key regulators of EO biosynthesis, influencing precursor formation and terpene diversity (Singh et al. 2024). However, transcriptomics further reveals the gene expression dynamics, proteomics and metabolomics provide valuable insights into enzyme functionality and metabolite composition, collectively offering a comprehensive perspective on EO production. Therefore, suitable integration of omics technologies enables the identification of regulatory pathways, enhancement of EO yield, with deeper understanding of metabolic flux in plants (Li et al. 2024).

Transcriptome analysis is the study of the complete set of RNA transcripts produced by the genome under specific circumstances or in a specific cell. It provides a snapshot of gene regulation and expression at a particular instance, allowing researchers to understand which genes are active, how their expression levels change in different conditions, and how they contribute to various biologic processes. Transcriptome analysis has identified the differential genes responsible for EO content in callus and tissue culture seedlings of *Lavandula angustifolia.* The study offers deeper insights into the regulatory genes involved in terpenoid biosynthesis and brassinosteroid signal transduction, contributing to the genetic improvement of *Lavandula angustifolia* (Cheng et al. 2024).

Although tissue culture technique could be effectively explored for EO production, it poses several challenges. Since these cultures are highly prone to microbial contamination maintaining aseptic condition throughout the production cycle may be cumbersome thereby increasing operational complexity while scaling up. In addition, scale up challenges with respect to nutrient distribution, oxygen transfer in bioreactors may be obvious. Moreover, the need for dedicated bioreactor designs, unremitting monitoring, and sterilization processes increases production costs could limit its commercial feasibility. Advancing research in this sector, supported by government funding opportunities, could help develop cost-effective bioreactor designs and optimized culture conditions, making large-scale essential oil production more feasible. Strategic investments in biotechnology and sustainable extraction methods could enhance commercial viability and encourage eco-friendly production practices.

### Role of characterization in essential oil quality checks

Ensuring the purity of EOs, free from any adulteration, has become a critical concern for aromatherapy practitioners, individuals in the cosmetic industry, and consumers alike (Cuchet et al. 2023). The demand for EOs across various sectors has led to unethical practices, such as blending them with cheaper oils, synthetic compounds, fragrance compounds, plasticizers, and other undesirable substances. These practices prioritize profit over the potential long-term health risks they pose.

To combat these practices and ensure the utmost value from EOs, it is crucial to establish high standards in their production and recovery processes. A combination of physical and analytical techniques is extensively employed to accurately assess the quality of oils, maintain transparency in testing methods, and establish a consistent functional fingerprint (Aatiaoui et al. 2022). Organoleptic evaluation by sensory experts helps detect slight odor variations, allowing for early problem identification before using analytical methods. For instance, sensory experts from the Food Quality and Safety research group assessed parsley samples, rating odor intensity on a scale from 0 to 10 (El-Zaeddi et al. 2016).

Physical tests, such as specific gravity measurements and refractive index determination using prisms, are commonly performed. The optical rotation of EOs is measured using a polarimeter, indicating whether the rotation is levorotatory (negative values) or dextrorotatory (positive values). Gas chromatography (GC) and Fourier-transform infrared spectroscopy (FTIR) are employed to confirm the presence of individual volatile compounds, while MS data provides information on their percentage presence. These quality checks offer valuable insights for refining the oil production process and maximizing profits (Spinozzi et al. 2023).

In recent times, the analysis of pesticide and heavy metal residues in EOs has become a common practice. Strong acids are used for digestion, and plasma is employed to ionize elements in inductively coupled plasma mass spectrometry (ICP-MS) (Jurowski et al. 2022). This analysis is crucial as these substances can co-extract during the extraction process, potentially leading to their bio-magnification in the oils and posing a threat to human health. Therefore, thorough evaluation of EOs using sophisticated techniques and proper labeling is essential for the benefit of users. Overview of various EO sources, extraction methods used and most common analytical techniques used for identification of key compounds is shown in Table 3.

**Table 3:** Overview of essential oil sources: extraction methods, analysis techniques, and key identified compounds

Source	Plant part used	Method of extraction	Analysis	Key identified compounds	Country	References
*Eucalyptus globulus*	Leaves		GC GC/MS	• *1, 8-cineole (63.1%) *p-cimene (7.7%) *a-pinene (7.3%) *a-limonene (6.9%)	Europe	Čmiková et al. (2023)
Centaurea vandasii Velen	Flowered aerial parts	Hydrodistillation	GC/MS	• *Hexadecanoic acid (18.3%) *Tetradecanoic acid (13.8%) *Caryophyllene oxide (12.1%) *Germacrene D (8.4%)	Bulgaria	Bancheva et al. (2021)
*Calamintha*	Leaves	Hydrodistillation	GC–MS FTIR	• *Menthol, cis-1, 3, cis-1, 4 (53.46%) *2-Isopropyl-5-methylcyclohexanone (3.66%) *p-Menthan-3 one (18.25%) *Iso-menthan-3-one (11.70%) *D-Limonene (4.06%)	Oriental of Morocco	Aatiaoui et al. (2022)
*Carlina acaulis*	Roots	Microwave-assisted hydrodistillation	GC–MS Density Refractive Index	• *Thymol *p-cymene *γ-terpinene	Italy	Spinozzi et al. (2023)
*Gynoxys rugulosa* Muschl. (Asteraceae)	Leaves	Steam distillation	GC–MS GC–FID	• *α-pinene (5.3–6.0%) *(E)-β-caryophyllene (2.4–2.8%) *α-humulene (3.0–3.2%) *germacrene D (4.9–6.5%) *δ-cadinene (2.2–2.3%) *caryophyllene oxide (1.6–2.2%) *α-cadinol (3.8–4.4%) *1-nonadecanol (1.7–1.9%) *1-eicosanol (0.9–1.2%) *n-tricosane (3.3–3.4%) *1-heneicosanol (4.5–5.8%) *n-pentacosane (5.8–7.1%) *1-tricosanol (4.0–4.5%) *n-heptacosane (3.0–3.5%)	Southern Ecuador	Maldonado et al. (2023)
*Cinnamomum loureirii* Nees	Bark and leaves		GC–MS GC–EAG	• *Cinnamyl acetate *trans-cinnamaldehyde	China	Xing et al. (2004)

*GC* Gas chromatography, *GC/MS* Gas chromatography/mass spectrometry, *FTIR* Fourier transform infrared spectroscopy, *GC-FID* Gas chromatography flame ionization detector, *GC-EAG* gas chromatography-electroantennography)

### .

## Regulatory guidelines for essential oils: production, sale, and usage

EOs necessitates robust regulatory measures to guarantee their safe manufacturing and sales. The involvement of regulatory authorities is dominant in upholding product authenticity. Prominent international organizations, such as the Food and Drug Administration (FDA), play a crucial role in safeguarding consumers from contemporary misleading claims made by companies regarding the potential of EOs. The FDA’s primary focus lies in ensuring consumer protection by verifying product safety, promoting accurate labeling, and providing comprehensive information on safety precautions and proper usage methods. By persistently monitoring and regulating the industry, these regulatory bodies contribute to building consumer trust and transparency within the EOs market. Under FDA regulations for aromatherapy, EOs are classified as cosmetics, drugs, or both. If an EO product is intended for therapeutic use and makes strong claims like muscle relaxation or treating depression, it falls under the drug category. However, obtaining FDA approval for EOs as drugs is a cumbersome, costly, and time-consuming process. Therefore, EO manufacturers often refine their claims to avoid classification as drugs (Osaili et al. 2023**).** To curb overexploitation and illegal trade of endangered plant species which are economically valuable, it is imperative to enforce strict regulations such as the Convention on International Trade in Endangered Species of Wild Fauna and Flora (CITES) (Garrison 1994). The motto of these regulations is only to safeguard the decline of endangered plant species. By implementing CITES, governments and authorities can efficiently curb the economic motivations driving the exploitation of endangered plants. In addition, enforcing CITES is challenging due to activities such as illegal trade, lack of standardized monitoring approaches, mislabeling, and difficulties in distinguishing legally cultivated species from wild-harvested ones. In addition, national and international standards for EOs include ISO standards like ISO 9235:2013, which defines terms for natural aromatic raw materials (Api et al. 2022). ISO 3515:2002 specifies guidelines on oil using steam distillation (Székely-Szentmiklósi et al. 2024), and ISO 11024-1:1998 which cover general guidelines for EO analysis (Chromatographic profiles) (Baycheva et al. 2889).

To ensure benign production and marketing of EOs in the Indian market, it is essential to adhere to both international based regulations, such as FDA guidelines, as well as countries own laws established by numerous regulatory bodies. These include the Food Safety Standard Authority of India (FSSAI), the State Ayurvedic License Authority (AYUSH), the Central Insecticidal Board and Registration Committee (CIB & RC), the State Licensing Authority of the Central Drug Standard Control Organization (CDCSO), and the Directorate General of Foreign Trade (DGFT). Obtaining an initial manufacturing license, based on the production scale, requires approval from the state licensing authority. Adhering to good manufacturing practices is essential, encompassing activities such as maintaining facility hygiene, equipment maintenance, documentation, and quality control. Labeling and packaging of EOs are crucial requirements, necessitating comprehensive information on the product’s name, manufacturing details, batch number, expiration date, and any usage or storage conditions. If the product contains alcohol, permission from the state excise department is required. In addition, if the product possesses insecticidal properties, approval from CIB & RC is necessary. Approval from FSSAI is mandatory if the product is used in food, and if it is used in cosmetics, approval from CDCSO is required.

## Business innovations in essential oils usage

The utilization of EOs has undergone a significant transformation from their traditional application solely in the healthcare sector. Their exceptional benefits have led to a paradigm shift in their usage across various sectors. This section provides an overview of the innovative applications of EOs, such as their role as preservatives to extend shelf life and their use in aromatherapy for pain reduction. In addition, recent advancements in these fields are discussed to showcase the latest developments and updates.

### Food preservation

Over the years, numerous innovative applications of EOs have been investigated, including their potential as environmentally friendly substitutes for toxic chemical preservatives such as nitrites or nitrates. Researchers explored the effectiveness of a combination of EOs derived from *Piper nigrum, Thymus vulgaris*, and *Syzigium aromaticum* in preserving pork sausages at concentrations ranging from 300 to 5000 ppm. The results demonstrated that the growth of *E. coli* ATCC25922, *S. aureus* NCTC10652, and *S. enteritidis* was completely inhibited within 3 days, significantly prolonging the shelf life of the sausages. Moreover, the sensory characteristics of the sausages were also enhanced (Leroy and Jean-Justin 2023). An experiment was conducted to assess the effectiveness of incorporating oregano EOs into mesoporous silica film packaging for extending the shelf life of edible mushrooms (*Agaricus bisporus*). The mushrooms were stored under controlled conditions of 4 ± 1 °C, and after 12 days, their hardness and nutrient contents were evaluated. The results revealed that the mushrooms packed with film containing oregano EOs exhibited delayed aging, reduced weight loss, and minimal respiration rate, which contributed to the preservation of their shelf life. Furthermore, the innovative packaging materials demonstrated promising outcomes by minimizing the rate of nutrient loss (Yan et al. 2023).

Furthermore, investigations have been conducted into the utilization of plant volatiles for preserving post-harvest blueberries, given that approximately 80% of these fruits are susceptible to fungal infections. EOs derived from Monarda didyma L, as well as their nano-emulsions, exhibited significant antifungal activity against Alternaria sp. and Colletotrichum sp. at minimal concentrations of 1–4 µL/ml. comparatively, the nano-emulsions demonstrated superior results compared to the plain EOs. Hence, the potential of employing plant EOs as nano-emulsions warrants further exploration as a promising antifungal agent for post-harvest blueberry preservation. This approach has the potential to extend shelf life and maximize profits (Zhang et al. 2023).

The inconsistent release kinetics of EOs embedded in packaging materials during various stages of food preservation have posed a significant challenge. In order to address this issue, researchers have explored the application of pH-sensitive dynamic release mechanics. This approach involves utilizing cinnamon EOs as the core and L100 polymer as the shell, achieved through the coaxial electrospinning technique, specifically for meat preservation. As pH levels in meat can fluctuate due to microbial activity during storage, leveraging these pH changes enables the active packaging material to transition from a solid to a liquid state, thereby activating its antimicrobial properties. By harnessing the natural pH variations, this innovative method allows for efficient preservation of meat by effectively responding to the specific conditions it encounters throughout storage (Zhang et al. 2023).

### Aromatherapy

Aromatherapy, utilizing EOs, is increasingly recognized in the global market due to the rise in pain-related issues resulting from today’s stressful lifestyle. Therapists and spas are witnessing a significant surge in demand for EOs, as they have been proven effective in reducing pain.

In a randomized sampling study, the effectiveness of rose EOs in addressing anxiety and sleep disorders among burn patients was explored. The participants were divided into two groups. The first group received routine care along with 5 drops of 40% rose EO for 3 consecutive nights, while the control group received 5 drops of distilled water as a placebo. Both groups were assessed for anxiety and sleep quality before and after the treatment. The results revealed a significant improvement in sleep quality scores among patients who received rose EOs as aromatherapy. This highlights the potential of utilizing rose EOs as a positive approach for treating anxiety and sleep disorders (Mokhtari et al. 2023). In a three-week randomized controlled trial, rheumatoid arthritis patients with joint pains and sleep disturbances received aromatherapy massage followed by video streaming three times a week. The intervention group was treated with 5% EOs, the placebo group received sweet almond oil, and the control group had no intervention. Pain, sleep quality, and sleepiness were assessed using standardized scales. Results showed that aromatherapy significantly improved sleep quality in the intervention group. However, more research is needed to fully understand the pain-relieving potential of EOs for rheumatoid arthritis patients (Lu et al. 2023).

The aforementioned case studies provide compelling evidence of the promising potential of EOs in aromatherapy for pain relief. As a result, the demand for EOs in the market is expected to increase. This growing demand necessitates the adoption of modern and innovative approaches, such as AI and ML, to assist farmers in optimizing the growth rate of EO-producing plants. In addition, these technologies can aid in identifying suitable processing techniques that maximize EO yields. Furthermore, automated systems can be implemented to ensure the quality of oils and provide valuable information on various quality aspects. By embracing these advanced methods, the EO industry can thrive and meet the evolving market demands.

## Role of AI and ML

In recent years, it is witnessed that digital technology has a major influence on business innovations (Sircar et al. 2021) because it converges the boundaries between the physical and biologic field by using ML, AI and robotics. This technological development is entering into all domains and the EO extraction is not an exception. Hence, the growth of technology has influenced the oil industry in the past few years. In the present section, an overview on usability and suitability of ML for analyzing chemical composition, activities and classification of EOs are provided.

**Fig. 7:**
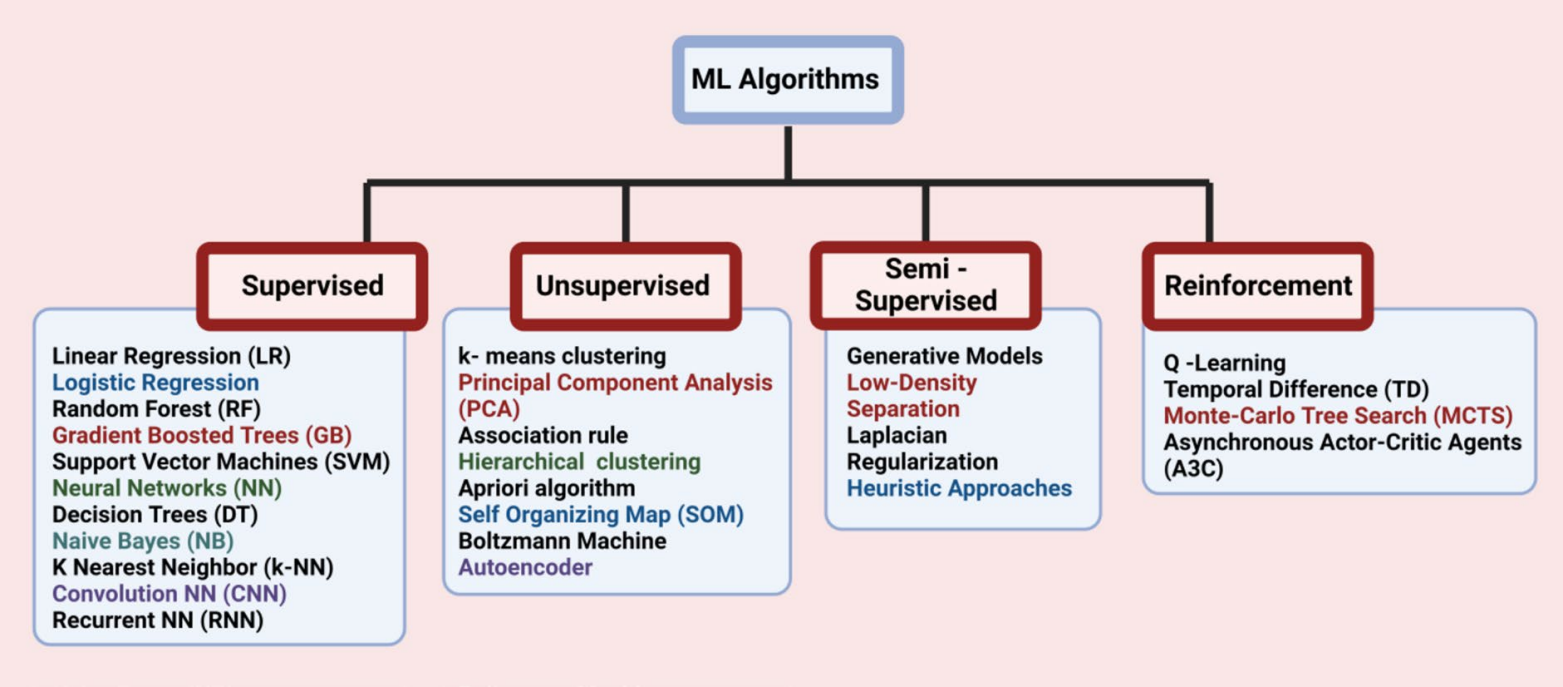
Various types of ML algorithms and popularly used algorithms

ML is a wing of AI that utilizes algorithms and information in the form of data to learn and predict. This enables computers to form opinions by making decisions. However, there are different types of ML algorithms, data types used for learning and the type of prediction to be made. Various types of ML algorithms and popularly used algorithms under each type are shown in Fig. 7. Each type of the algorithm has its own computational complexity and resource requirement. Hence, researchers need to wisely select the algorithms based on availability of the data and resources.

**Fig. 8:**
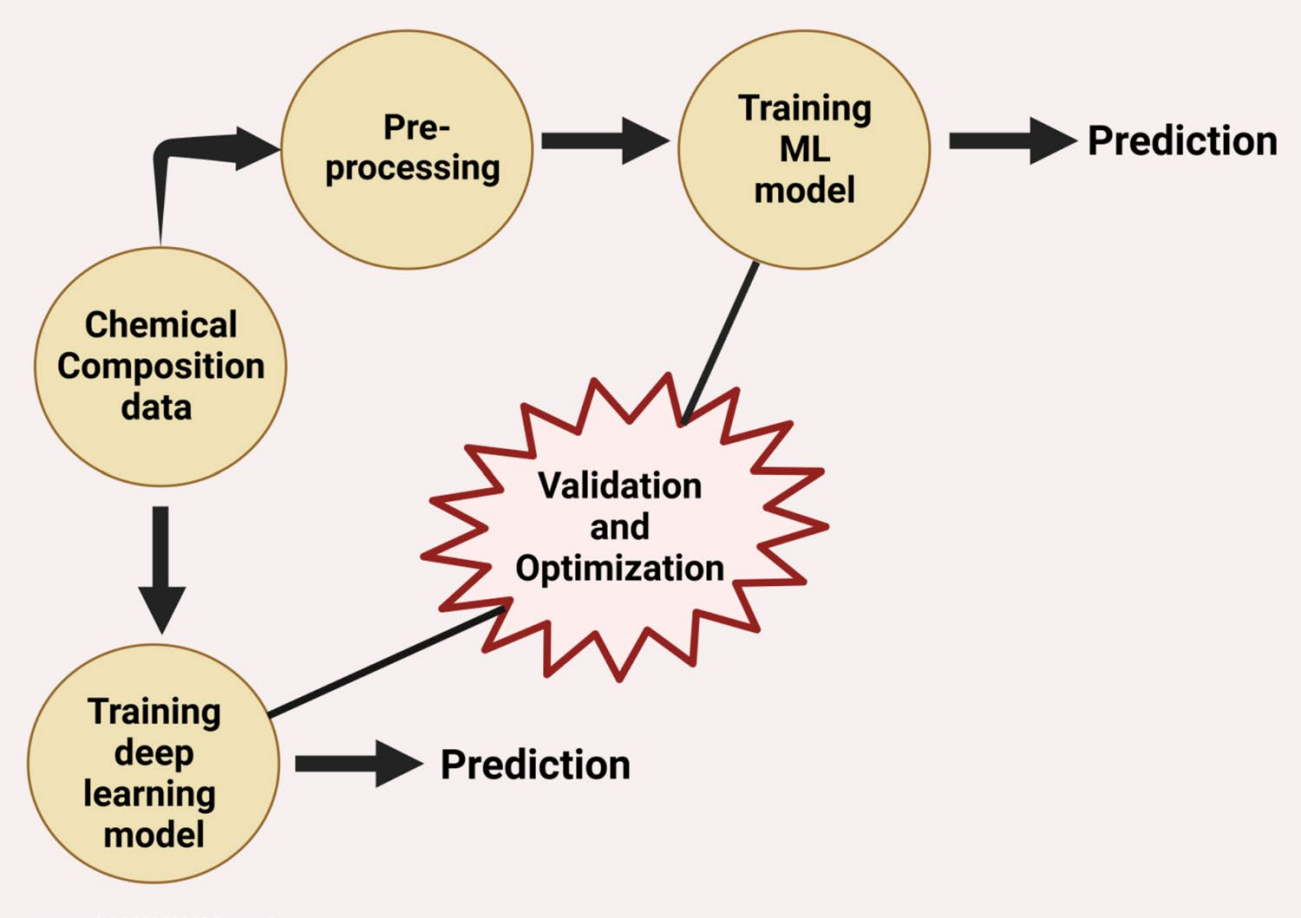
General flow of ML approach

EO plants are popular for their bioactivities such as antiviral, antiseptics, antiwormal, antibacterial, anti-inflammatory, antioxidant, antitumor, etc. (Beg et al. 2017). These activities vary from plant to plant based on chemical composition and geographic location. Therefore, there is a need for developing an automated model that is computationally efficient to handle the variations for predicting and classifying the biologic activities of EO plants. However, major challenge would involve having a sufficient number of descriptive data to design an efficient ML model. In addition, it is evident from literature reports that noise in data, biases in datasets, or the need for feature selection in ML models could affect model outcomes (Jalali-Heravi and Parastar 2011). Further, the data are processed and used for training a ML model for the prediction as depicted in Fig. 8.

Predictive models can be designed using two approaches namely conventional ML referred to as ML in this paper and DL. Rigorous training by feeding a variety of data is essential to design an efficient model in both the approaches. This involves validation and optimization of the models while training as shown in the diagram. However, use of raw data is not suggested in the case of training ML. Hence, pre-processing techniques such as data cleaning, normalization, noise removal, etc. may be carried out for better efficiency of the ML models. In the DL approach raw data can be directly used for training. However, data normalization cannot be avoided.

It is a well-known fact that EO is a combination of numerous chemical compounds. Analysis of such chemical compound patterns can be automated using ML which may lead to the determination of bioactivities approaches (de Menezes et al. 2022). Also, based on the determined bioactivities, a decision can be easily made whether to use the EOs in pharmaceuticals industries, food products, cosmetics or as perfumes (Turek and Stintzing 2013). Hence, such attempts are made by many research groups and can be found in a systematic review on EOs reporting 585 articles across 80 countries including 290 plant genera, 150 chemical compounds, 30 genera of bacteria, and ten genera of fungi (Galvan et al. 2021). In addition, ML methods like K mean clustering and decision tree (DT) have been successfully utilized for predicting the components of *Laurus nobilis L.* EO. It was evident that decision tree algorithm and K mean clustering classification achieved an accuracy rate of 96.23% and 97.83%, respectively. This confirms the potential of ML algorithms as essential guides for research related to EOs (Uzun and Saltan 2024). Recently, ML classification algorithms like SVM, RF, Gradient Boosting (GB), DTs, and K-Nearest Neighbors have been utilized effectively identifying key components in EOs with high antibacterial activity against *Acinetobacter baumannii* with enhanced biologic efficacy and low cytotoxicity. This paves way for clinical application of EOs research with advances in technology (Astolfi et al. 2025). Nutraceutical potential of EOs for enhanced wellbeing of humans is also explored recently due to rich availability of bioactive compounds in natural form. This could be effectively exploited for value addition in functional foods and dietary supplements for promoting health and preventing chronic diseases (Olalere et al. 2024). In addition, antimicrobial properties of EOs are exploited against potential infections. Bacteria are one among the potent organisms which can cause biofilm associated infections. Intensive and exhaustive use of antimicrobial drugs to fight any infections may lead to antibiotic resistant bacterial strains in the human body. Hence, it is essential to investigate the use of natural derived compounds such as herbs or EOs to prevent the formation of such biofilm. However, manual investigation for such issues could be laborious and the solution lies in using ML driven approach. There are documentations in the literature for employing ML algorithms to determine antibiofilm activities of EOs against *Staphylococcus (S. aureus)* species, a versatile pathogen (Patsilinakos et al. 2019; Ragno et al. 2020; Yabuuchi et al. 2023). Biologic activity of EO against both *S. aureus* and *E.coli* has been investigated using RF algorithm reporting promising results (Barros et al. 2018). The overuse of standard antibiotics has led to increased antibiotic resistance, necessitating the search for alternatives. Recent studies have focused on using AI to quickly identify potential plant sources for extracting EOs that can control antimicrobial-resistant strains, thereby reducing mortality.

*Pseudomonas aeruginosa*, a type of bacteria that causes infections in the blood and lungs can cause infection is hospital patients after surgery. An investigation using ML algorithms on antimicrobial and antibiofilm activities of EO extracted from mediterranean plants against the bacteria confirmed the suitability of ML models in the present field (Artini et al. 2018). Naturally, bioactivity of EO is based on chemical structure. A research group (El-Attar et al. 2020) performed an experimental based analysis using these chemical structures to train CNN algorithms for classification of various types of bioactivities such as antiviral, antiwormal, antimicrobial, antioxidant, antifungal, etc. A relation between chemical compositions and bioactivities of various EO is modeled using RF algorithm to generalize and enhance the main chemical composition for the activity was also tried (Ragno et al. 2021). This study focused on the classification of bioactivity based on chemical composition thereby bringing out the most prominent chemical property required for the type of bioactivity. Normally EOs is extracted from herbs, roots and seeds. It is essential to check the quality of herbs or leaves to obtain good quality EO. Toward this direction, quality estimation of *Mentha aquatica* L. herb was carried out accounting color features, moisture ratio, EO content and major constituents employing NN (Taheri-Garavand et al. 2021). A similar approach can be found for estimating EO yield in turmeric from the air dried roots (Akbar et al. 2018). Healthiness of any crop and its extracts are highly dependent on the quality of seed and seedling. For maximum yield it is necessary to regularly monitor the seed cycle. However, it is a tedious task to observe every seed that is sowed beneath. Recent study reported an image based DL algorithm for automated monitoring of seed cycle of EO plants (Samiei et al. 2020). Further, after the extraction of EO, quality checking is mandatory before releasing it to the market. smartphone-based miniaturized Raman spectrometer for quality estimation of EO have been tried (Lebanov and Paull 2021).

The aforementioned state-of-the-art studies have opened new doors for the researchers in the field of EOs and the overview of the methods is provided in Table 4. However, rigorous experimentations and validations are required for using them practically in the real fields such that statistical evaluation of ML prediction maps the manual prediction. Such algorithms could be used in real-time applications. Also, there are many benefits of EOs that are currently being investigated manually in various fields such as naturotherapy, aromatherapy, and replacement for chemical pesticides and so on. Application of ML could be driven to these areas for developing assisting devices.

**Table 4:** Overview of ML driven state-of-the-art methods

ML algorithm	EO extracts	Investigation carried out	References
SOM and RF	Cuban plants	Antiprotozoal activity: antiplasmodial, antileishmanial, antitrypanosoma	Menezes et al. (2022)
PCA, PLS-DA	*Calamintha Nepeta* (CNEO), *Foeniculum vulgare* (FVEO) and *Ridolfia Segetum* (RSEO) plants	Active, non-active, toxic, non-toxic	Sabatino et al. (2020)
RF, GB, SV, LR, DT and KNN	61 EOs	Antibacterial activity on *S. aureus* strains	Papa et al. 2020)
PCA, LR, SVM	89 EOs extracted from 3 different plants: *Ridolfia segetum Moris* (RS); (FVEOs) from *Foeniculum vulgare Miller* (FV) and) from *Calamintha nepeta*	Antibiofilm Activities against *Staphylococcus aureus*	Patsilinakos et al. (2019)
Clustering	6 types EOs	Antimicrobial activity on *P. aeruginosa* and *S. aureus*	Ragno et al. (2020)
SVM, RF, DNN	*Lindera triloba* and *Cinnamomum sieboldii*	Antimicrobial activity against *Staphylococcus aureus*	Yabuuchi et al. (2023)
PCA, GB	3 types of EOs extracted from Mediterranean plants	Biofilm formation: Activity against Pseudomonas aeruginosa	Artini et al. (2018)
CNN, NN	120 plants, endemic Egyptian plants	Prediction of different bioactivities	El-Attar et al. (2020)
LR, SVM, GB, k-NN, RF	61 EOs (used dataset from Papa et al. (2020))	Inhibition activity against *Microsporum spp*: Antidermatophyte Activity	Ragno et al. 2021)
RF	Secondary metabolites isolated from the *Solanum genus*	Biologic activities against pathogenic bacterium: methicillin-resistant *S. aureus* (MRSA) and* E. coli*	Barros et al. (2018)
			Taheri-Garavand et al. (2021)
ANN	Water mint (*Mentha aquatica* L.)	Herb quality estimation	Samiei et al. (2020)
CNN	Two species of *red clover* and *alfalfa*	Seedling monitoring	Akbar et al. (2018)
ANN	Turmeric root (air dried)	EO yield in turmeric (*Curcuma longa L*.)	Lebanov and Paull (2021)
RF and PLS-DA	–	EO into pure and adulterated	

*SOM* Self organized map, *RF* Random Forest, *PCA* Principal component analysis, *PLS-DA* Partial least squares discriminant analysis, *GB* Gradient Boosting, *SVM* Support Vector Machine, *LR* Logistic Regression, *DT* Decision Tree, *KNN* k-Nearest Neighbors, *SVM* Support Vector Machine, *NN* Neural network, *DNN* Deep Neural Network, *CNN* Convolutional Neural Network, *ANN* Artificial neural network

AI has the potential to revolutionize the EO industry through automated monitoring, provided that challenges related to EO datasets are carefully managed to meet training and testing requirements. Recent advancements in the use of AI for quality control of EOs have been significant. Studies have shown the application of AI techniques such as artificial neural networks (ANNs) for classifying high and low-quality agarwood EOs based on specific compounds (Mahabob et al. 2022; Mahabob et al. 2022). Furthermore, AI models such as multi-layer perceptron (MLP) and ANNs have been created to reliably quantify the relationship between input variables and chemical extraction output in EO steam distillation processes, facilitating large-scale production control (Cordero et al. 2022). Moreover, integrating AI with advanced analytical techniques such as comprehensive two-dimensional gas chromatography coupled with time-of-flight mass spectrometry (GC × GC-TOF MS) has made it possible to predict key aroma signatures in food products. This innovation highlights the potential for detecting odor patterns and performing sensory evaluations without human olfaction, especially in high-quality olive oil production (Fajardo Muñoz et al. 2023). These advancements highlight the growing role of AI in enhancing the quality control processes of EOs through automation and advanced analytical methodologies. AI systems can thus continuously monitor the quality of EOs by analyzing various parameters in real-time, such as chemical composition and purity. And ML algorithms can detect anomalies and ensure consistency in product quality.

AI also plays a crucial role in revolutionizing Predictive Maintenance (PdM) practices in the oil and gas industry, including EO facilities. By leveraging AI-driven analytics and ML algorithms, companies can predict equipment failures with unprecedented accuracy, enabling proactive maintenance interventions and minimizing costly downtime (Chuka Anthony Arinze 2024). AI facilitates the analysis of extensive data streams from sensors and equipment, allowing for precise failure predictions and cost-effective maintenance actions, ultimately optimizing system reliability and performance (Nadaf 2024). ML techniques in PdM aid in reducing unscheduled downtime, maintenance expenses, and enhances operational efficiency by identifying and addressing potential equipment issues in advance (Narkarunai Arasu Malaiyappan et al. 2024). The integration of AI in PdM marks a paradigm shift, offering a proactive approach to asset management and operational excellence. AI can contribute to sustainable development in classification methods used by major E-commerce companies, enabling the extraction of new knowledge datasets through Natural Language Inference (NLI) techniques (Shao et al. 2023). Unquestionably, the integration of AI in consumer insights for EO usage offers a comprehensive and data-driven approach to understanding consumer behavior and preferences (Nunes et al. 2023). Therefore, future research trends which focus on use of AI for the below points could be a boon for EO commercialization.

I. Understanding consumer needs and suitable customization of products
II. Computational chemistry for design and synthesis of novel EO compounds with targeted therapeutic effects.
III. Integration of AI with IoT devices for real-time monitoring and optimization of EO production processes.
IV. Predictive models for sustainable harvesting and reducing waste.
V. Automated documentation and real-time compliance monitoring.
VI. Exploration of new plant species for commercial EO extraction.

## Conclusions and future visions

In conclusion, this comprehensive guide on unlocking the potential of EOs has shed light on the significant advancements in recovery techniques, modern innovations, regulatory frameworks, and AI integration in the field. By emphasizing the importance of quality control and exploring various extraction methods and emerging technologies, the guide has highlighted the possibilities for refining the purity and effectiveness of EOs. Moreover, the discussion on modern innovations and their applications in industries like food and personal care has showcased the expanding scope and potential of EOs s. Finally, the exploration of AI integration has demonstrated the transformative impact of technology in enhancing EO research, development, and decision-making processes. Exploring unexplored benefits of EOs in various fields presents exciting prospects, and ML integration can foster supportive devices for research and practical applications. Overall, this guide serves as a valuable resource for leveraging the vast potential of EOs across diverse domains of food preservation and aromatherapy.

## Data Availability

Data will be made available on considerable request.
